# Dietary Sugar Intake and Its Association with Obesity in Children and Adolescents †

**DOI:** 10.3390/children8080676

**Published:** 2021-08-03

**Authors:** Emmanuella Magriplis, George Michas, Evgenia Petridi, George P. Chrousos, Eleftheria Roma, Vassiliki Benetou, Nikos Cholopoulos, Renata Micha, Demosthenes Panagiotakos, Antonis Zampelas

**Affiliations:** 1 Department of Food Science and Human Nutrition, Agricultural University of Athens, Iera Odos 75, 118 55 Athens, Greece; gv.michas@gmail.com (G.M.); azampelas@aua.gr (A.Z.); 2 Department of Life and Health Sciences, University of Nicosia, Makedonitisas Avenue, Nicosia CY1700, Cyprus; evipetridi@hotmail.com; 3 First Department of Pediatrics, Medical School, National and Kapodistrian University of Athens, Mikras Asias 75, 115 27 Athens, Greece; chrousos@gmail.com (G.P.C.); roma2el@otenet.gr (E.R.); 4 Department of Hygiene, Epidemiology and Medical Statistics, School of Medicine, National and Kapodistrian University of Athens, 115-27 Athens, Greece; vbenetou@med.uoa.gr; 5 Department of Medicine, School of Health Sciences, Aristotle University of Thessaloniki, University Campus, 54 124 Thessaloniki, Greece; n.cholopoulos@gmail.com; 6 Department of Food Science & Human Nutrition, University of Thessaly, 382 21 Volos, Greece; Renata.Micha@tufts.edu; 7 Friedman School of Nutrition Science and Policy, Tufts University, Boston, MA 02155, USA; 8 Department of Nutrition and Dietetics, School of Health Science and Education, Harokopio University, Athens, Eleftheriou Venizelou 70, 176 76 Athens, Greece; dbpanag@hua.gr

**Keywords:** total sugars, added sugars, children, adolescents, overweight and obesity, dietary intake

## Abstract

Sugar intake has been associated with increased prevalence of childhood overweight/obesity; however, results remain controversial. The aim of this study was to examine the probability of overweight/obesity with higher sugar intakes, accounting for other dietary intakes. Data from 1165 children and adolescents aged ≥2–18 years (66.8% males) enrolled in the Hellenic National Nutrition and Health Survey (HNNHS) were used; specifically, 781 children aged 2–11 years and 384 adolescents 12–18 years. Total and added sugar intake were assessed using two 24 h recalls (24 hR). Foods were categorized into specific food groups to evaluate the main foods contributing to intakes. A significant proportion of children (18.7%) and adolescents (24.5%) exceeded the recommended cut-off of 10% of total energy intake from added sugars. Sweets (29.8%) and processed/refined grains and cereals (19.1%) were the main sources of added sugars in both age groups, while in adolescents, the third main contributor was sugar-sweetened beverages (20.6%). Being overweight or obese was 2.57 (*p* = 0.002) and 1.77 (*p* = 0.047) times more likely for intakes ≥10% of total energy from added sugars compared to less <10%, when accounting for food groups and macronutrient intakes, respectively. The predicted probability of becoming obese was also significant with higher total and added-sugar consumption. We conclude that high consumption of added sugars increased the probability for overweight/obesity among youth, irrespectively of other dietary or macronutrient intakes.

## 1. Introduction

The prevalence of obesity nearly tripled from 4% in 1975 to over 18% in 2016 worldwide [1] with 124 million children and adolescents aged 5–19 years old being classified as obese and 213 million as overweight in 2017 [2]. These numbers rank childhood obesity as one of the major public health issues of the 21st century [3] as it is generally associated with obesity in adulthood. Childhood obesity is also associated with premature onset of cardiovascular disease risk factors such as hypertension, dyslipidemia, insulin resistance, and glucose intolerance [4].

Sugar consumption has been associated with reduced diet quality, increased energy intake [5], and with increased prevalence of obesity. These findings, however, remain controversial, especially with regards to energy consumption [6,7]. The controversy is further enhanced, by recent findings from a large national US survey, which showed that total sugar intake among adults had decreased in the past years, to levels consumed in 1975, while the prevalence of obesity remained increased [8]. Interestingly, in children and adolescents, however, 16% of their total energy intake was obtained from added sugars, which is 6% higher than the current recommendations [8]. Although most public health professionals agree on limiting added sugars, this is mostly recommended in an attempt to minimize empty calory intake [9], and increase nutrient density; it is not based on sufficient evidence for obesity reduction, since data remain inconclusive. The World Health Organization (WHO), however, strongly emphasizes on reducing free sugars intake to <10% of total energy intake, for preventing and controlling excess body weight [1]. Reduction to <5% has been acknowledged as a recommendation to prevent dental caries alone [1].

The association, therefore, between obesity rates and added sugars and/or total sugar intake remains under investigation, especially among children. Several studies have investigated the association of sugar intake and weight status in children [10,11,12,13,14,15,16,17,18,19,20]. The majority of them have investigated potential effects of added sugars and SSB’s on Body Mass Index (BMI). Specifically, Wang et al. (2015), indicated that liquid but not solid added sugars were positively associated with higher BMI for age, BMI z-scores, and body fat, although both were associated with higher energy consumption [21]. Other studies showed that an increased intake of SSBs was associated with an increase in BMI in preschool and school aged children [10,11,12,13]. In these studies, however, the main characteristic of the sampled children was frequent, often daily SSB consumption. A study that investigated the effect of SSB as well as artificially sweetened beverage consumption, on obesity, in school aged children in the UK, found a significant association in both cases [11]. This raises the question whether it is the whole dietary pattern, the sugar content consumed or the interaction of these, granted that artificially sweetened beverages do not provide added sugars or calories. Other studies have failed to observe an association between SSBs and body weight status in children [16,17,18,19,20]. Furthermore, studies investigating specifically total, added, or free sugars, depending on definition chosen, have been inconclusive with regards to their effect on weight in children and adolescents [14,15,16], despite the fact that in some populations sugar intakes were tabulated above the recommended levels among school aged children [14,17]. Finally, a recent systematic review has shown that dietary patterns which include sweets and other potential obesogenic foods may be contributing to childhood obesity via an interaction of foods and nutrients [22]. Consequently, an investigation of total sugars, SSBs, and added sugar intakes, in relation to dietary patterns, and their associations on childhood obesity may help to resolve these discrepancies.

Therefore, the purpose of this study was to investigate the association between total and added sugar intakes with overweight/obesity in children and adolescents, accounting for other dietary intakes, using nationally representative data. In addition, the study aimed to examine the major foods or food groups that contributed to total sugar intake by age group and sex.

## 2. Methods

### 2.1. Study Design and Population Sampling

The Hellenic National Nutrition and Health Survey (HNNHS) is a population-based survey launched between September 2013 and May 2015. The study was designed to assess the health and nutritional status of Greek residents, including children over 6 months and adults. Individuals residing in various institutions, members of the armed forces, those with progressed mental disabilities, and pregnant and lactating women were excluded. In more detail, HNNHS is a cross-sectional observational survey. Responders’ selection was performed with a random stratified design based on the 2011 census data. The sampling frame included a multi-stage stratified design and we aimed in achieving representativeness in six groups (0–19 years, 20–65 years, and 65+; in males and females), across four geographical regions of Greece. A total of 4574 individuals (42.5% males). A random selection of more than one individual per household was possible but no more than one individual from the same age group could be enrolled in the study. If households had children < 6 years of age, one (if more were present) was randomly selected to be included in the study, upon consent. For this study data from 1165 children and adolescents aged ≥2–18 years (66.8% males) were used. More details of the study have been published elsewhere [23].

### 2.2. Final Study Population and Factors Evaluated

For the purposes of this study, all children and adolescents ≥2–18 years that had provided at least one dietary 24 h recall (24 hR) were included. This resulted in a total sample of 1165 (66.8% males) of children (*n* = 781; aged 2–11 years) and adolescents (*n* = 384; aged 12–18 years). Children and adolescents that reported energy intakes >6000 kcal/ day (>25,080 kJ), were excluded to avoid extreme over-reporters. Due to the nature of the study sample, a specific value for extreme under-reporters was not set. A total of eight children, with no significant sex differences, had extremely overreported. The final sample consisted of 1165 children (≥2–11 years) and adolescents (≥12–18 years).

Parental or main guardian consent was signed for all underaged individuals, and by adolescents themselves if they were 18 years of age. The study was approved by the Ethics Committee of the Department of Food Science and Human Nutrition of the Agricultural University of Athens in 2013. The study was also approved by the Hellenic Data Protection Authority. It was conducted in accordance with the 1964 Helsinki declaration and its later amendments or comparable ethical standards. All members of the staff signed confidentiality agreements.

### 2.3. Data Collection and Dietary Assessment

Interpersonal Computer Assisted Personal Interview (CAPI) were performed by trained personnel, to obtain information on anthropometric, sociodemographic and lifestyle parameters. For dietary intake 24 hR’s were obtained using the Automated Multipass Method (AMPM) [24], in order to reduce reporting bias [25]. This method has been found by a recent systematic review to provide the most accurate energy consumption data for children aged 0–18 years when compared to doubly labelled water, using parents as proxy reporters [26]. The goal was to obtain two 24 hR, in non-consecutive days, 8–20 days apart: the first via interview and the second via phone. Parents or the primary guardian was used as a proxy responder for all children <12 years. Those ≥12 self-responded with the parent or guardian having an aiding action. Age-specific food atlases and specific grids and household volume measures such as cups, glasses, plates, and spoon sizes were used in order to achieve accurate portion sizes. All foods were disaggregated into specific food groups, 42 in total as the primary value as shown in Supplementary Table S1. For nutrient analysis the Nutrition Data System for Research (NDSR) (developed by the University of Minnesota) as well as Greek food composition tables for traditional Greek recipes (e.g., baklavas) [27] were used.

#### 2.3.1. Sugar Intake Assessment and Major Food Contributors

Total sugar, added sugar, as well as specific mono- and disaccharides were estimated for each food item reported in the 24 hR, using the Nutrition Data System for Research (NDSR) database, adapted with traditional and other newly marketed foods. The definition of the Food and Drug Administration (FDA) was used for total sugars: “the sum of all free mono and disaccharides” which would include glucose, fructose, galactose, and lactose as well as sucrose and maltose [7]. Moreover, the FDA defines added sugar as the “sugars and syrups that are added to foods during processing or preparation” excluding sugars naturally found in foods, such as fruits or dairy products. The sum of each mono- and di-saccharide was calculated and then the average intake per child per day was estimated and used for further analysis. Each of these intakes were further investigated in relation to children’s weight status.

Foods were also categorized into specific food groups differentiating SSBs from solid sugars, but also natural sources from processed foods containing added sugars. Major food groups contributing to total sugar intake were then investigated using specific methodological approaches. Specifically, the percentage contribution made by each food group to total- and added sugar was estimated for the total sample by sex, but also for children and adolescents, separately. This was calculated by dividing the total nutrient provided by a specific food group by the total nutrient provided by all food groups and multiplying this by 100 [28]. Children and adolescents were grouped based on % of total and added sugar intakes for further analysis. Specifically, for total sugars, the median of the population was used for comparative reasons, since to date no specific cut-off is recommended. For added sugars the 10% cutoff of total energy from added sugar consumption as recommended by the US Department of Health and Human Services and US Department of Agriculture [29].

#### 2.3.2. Other Nutritional Intakes

As per sugar intake, other macronutrients including total animal protein, plant protein, total fat, fiber (in grams), non-sugars carbohydrates, and energy were calculated for each child. In addition, specific food groups were derived, including non-milk dairy, plant protein, non-processed animal protein, processed animal protein, fruit, and vegetables. Grouping details can be seen in supplements (Supplementary Table S1), where the preliminary 42 food groups were regrouped for study purposes.

### 2.4. Anthropometric and Lifestyle Data

#### 2.4.1. Weight Status Assessment

Body weight and height were reported by the parent and guardian for children <12 of age and by the adolescents (≥12). For missing information on weight or height (*n* = 107 in total), multiple imputation was performed, using missing at random (MAR) process (STATA Corp LLC., College Station, TX, USA). Body Mass Index (BMI) was derived by body weight in kg divided by height in meters [2] and was used for the evaluation of weight status. All children and adolescence were categorized using the extended International Obesity Task Force (IOTF) tables. These tables correlate the children’s BMI by month after the 2nd year of age, to the respective adult’s BMI; these can be expressed as BMI centiles as well, for direct comparison with other child BMI references [30]. The descriptive table (Table 1) contains healthy weight children (none were found to be undernourished), overweight, and obese; overweight and obese children were grouped for further analysis, in order to have adequate power of analysis.

#### 2.4.2. Lifestyle and Other Variables

Details on other lifestyle and socioeconomic variables, such as physical activity, screen time, and parental educational level, known to be associated with adiposity, were also collected [11,31]. Specifically, physical activity was evaluated by two different questionnaires based on age groups (i) Pre-Physical Activity Questionnaire (PAQ) Home Version for children ≥2–<12 years old [32] and (ii) the International Physical Questionnaire–Adolescents (IPAQ-A), for those ≥12–<18 years old [33]. PAQ questionnaire estimated only specific activities performed over 7-days; IPAQ-A is also a 7-day recall instrument, used to estimate total metabolic equivalents (METS), based on information obtained on specific activities performed at leisure time, during transport, at school (physed and recess), and at home. These have been previously validated and PAQ-A specifically, has also been tested against doubly labelled water and was found to have a strong correlation with energy expenditure for grοups [34]. The measure of physical activity was estimated by multiplying the time spent with each activity with the corresponding MET-value per total minutes of activity as per various protocols [34]. Physical activity quartiles were derived to describe the children’s physical activity status.

Screen time was also obtained by calculating the average time spent in front of any type of screen, on weekday and weekends, excluding time spent using gaming platform that requires movement. Total screen time was categorized using the upper recommended level of 2 h per day.

The children’s or adolescent’s SES cannot differ from that of their parent hence parental educational level was used to categorize them accordingly and adjust in the models. If this was not performed systematic errors would prevail. The primary guardian’s educational level and type of profession were also accounted for, as socioeconomic proxies. Education was categorized into elementary (≤6 years), middle school (≥6–11 years), and higher level of education (≥12 years). Professional status was categorized as employed, unemployed and/or homeworker, and pension. The latter characterize mostly grandparents.

#### 2.4.3. Sensitivity Analysis

Extra statistical analysis was performed by reporting status in order to assess association differences and further decrease reporting error, since this was not controlled for. Specifically, it has been shown that dietary under-reporting prevalence is higher compared to over-reporting among children and adolescents [35]. Normal misreporters, including over- and under-reporters as identified based on sex, age, BMR, and IPAQ level, were therefore not excluded, in order to avoid a shift to the right which means a decrease in the estimated energy and macronutrients intake. A sensitivity analysis, however, per reporting status, was performed to investigate potential effect differences of added and total sugar on weight status. For this specific analysis, over- and under- reporters were identified by dividing total energy intake by calculated basal metabolic rate (BMR) using the Scholffield equation, including for IPAQ, and then using validated Goldberg cut-offs [36] for adolescents and those reported for children by age group and sex [35].

### 2.5. Statistical Analysis

Distribution plots were examined for continuous variables using k density kernels and P-P plots. All variables are summarized in total and by sex; numerical variables following normal distribution are depicted as means (±sd) and those skewed as medians and interquartile range (IQR) and categorical variables are presented as relative frequencies. Group differences were examined using parametric or non-parametric methods for numerical data, accordingly and chi-square test for categorical. Significance was assessed at α = 5%, using two-sided test (*p* < 0.05). Mixed effects logistic regression models were used to account for the skewed distribution of the data. The likelihood of being overweight/obese compared to healthy weight was explored as per (i) the median of total sugar intake (adjusted for energy) and (ii) added sugar intake above 10% (>10%) of total energy intake, and (iii) median intake of added sugar intake. Two models were used following preliminary analysis of intakes (i) food groups including animal- and plant- protein, non-milk dairy, fruit and vegetables; and (ii) macronutrient (fat, protein, other carbohydrates) and dietary fiber intakes. All models were adjusted for children’s’ lifestyle, activity level (IPAQ), total screen time, and the primary guardian’s socioeconomic status. Marginal effect sizes were also conducted to derive probability of overweight/obese status for higher total- and added- sugar intakes. The statistical software package STATA 12.0 was used for statistical calculations (STATA Corp LLC., College Station, TX, USA).

## 3. Results

**Figure 1:**
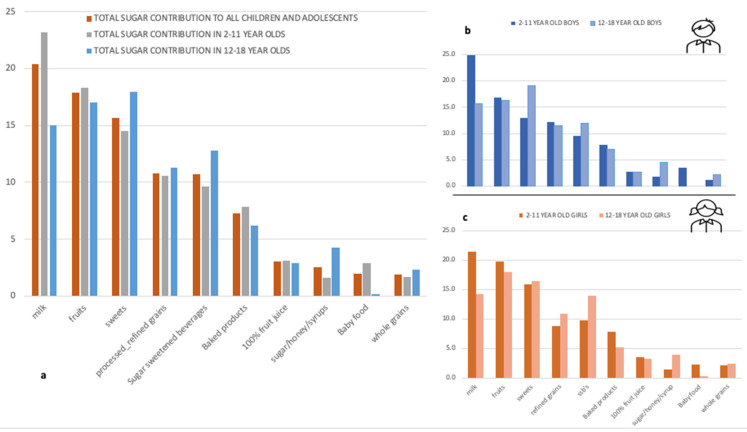
Total sugar contribution in children and adolescents, in total sample and by sex. (**a**). All children and adolescents; (**b**). boys; (**c**). girls.

Table 1 summarizes the anthropometric and lifestyle characteristics, the dietary intake of the children and the adolescents, as well as the main guardian sociodemographic information. In the present study, 10.6% of the children and 21.6% of the adolescents were overweight/obese. Median total sugar daily intake in relation to total energy consumption, was 15.6% (SE: 6.7), with a significantly higher consumption observed in children compared to adolescents (16.3% and 14.3% respectively; *p* < 0.001). The main food contributing to this higher intake was milk (Figure 1). The percentage of energy from added sugars was 9.5% in children and 5.5% in adolescents, with 18.7% of the children and 24.5% of the adolescents exceeding the 10% of total energy intake recommended upper limit (Table 1). As per basic lifestyle characteristics, physical activity levels differed between children and adolescents, with adolescents being more physically active. Overall, only 200 out of 781 children reported of performing a specific activity at least once a week (hence the median of “0”). In contrast although both children and adolescents spent more time in front of a screen than recommended (>2 h per day), children’s total screen time was higher than adolescents. Finally, primary guardian education level was between 6 and 12 years, and the majority of them were employed.

**Figure 2:**
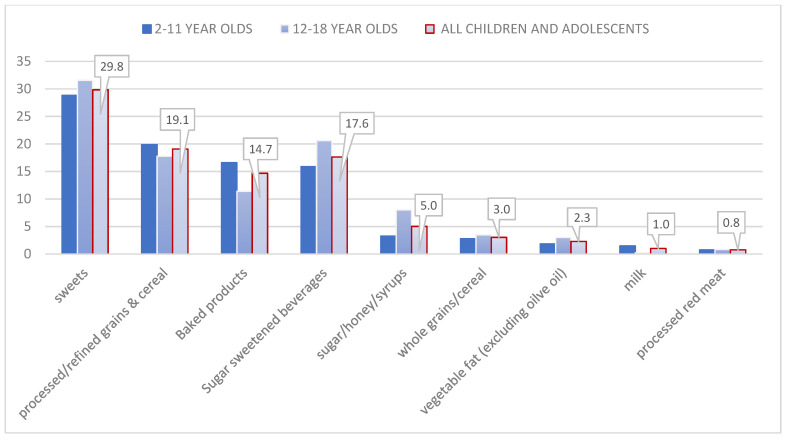
Main food groups contributors to added sugar intake in children and adolescents.

Food groups contribution for total and added sugars are shown in Figure 1 and Figure 2, respectively. For the total sample (Figure 1a), milk was the main contributor of total sugars (20.4%), followed by fruits (17.9%), sweets (15.7%), processed/refined grains (10.8%), and SSBs (10.7%). In total, these foods/food groups contributed 75.5% of total sugar intake. If baked products, that include cakes and cookies, are also accounted for and included, the contribution increased to almost 83.0%, 44.5% of which were from sources containing added sugars. When adolescents were differentiated from children, the main food group contributing to total sugars were sweets, especially among adolescent boys (Figure 1b), followed by fruits (17%), and the third main contributor was SSBs (12.8%); the latter being consistent in both boys and girls. The main food contributors in adolescent girls (Figure 1c) were fruits (18%) followed by sweets (16%), in comparison to boys which were sweets (18%), followed by fruits (16.2%). Overall, 100% juice contributed to 3% of total sugar intake and honey, added sugar, and syrups to 2.5%.

Figure 2 depicts the major food groups that contributed to added sugars, in total and by age group. The main two sources of added sugars were sweets (29.8%) and processed/refined grains and cereals (19.1%), in total and in both age groups. These were followed by SSBs (17.6%) in the total sample, due to high consumption among adolescents (20.6%), but was fourth in children (11.4%), following baked products (16.6%).

Table 2 summarizes the estimated mean energy, macronutrient, and selected recommended food group intakes, by level of added sugar consumption, according to current recommendations (≥10% compared to <10% of total calories). Total sugars were significantly lower, and total fat and animal protein were higher, in children and adolescents consuming <10% of energy from added sugars. Non-sugar carbohydrates were significantly higher whereas total fiber was lower, in children consuming <10% of energy from added sugars but did not differ in adolescents. No significant differences were found in vegetable protein, being low in both groups. Regarding specific recommended food groups, all were higher among children with greater added sugar intake, whereas in adolescents’ fruit and animal protein intake was significantly higher for those consuming <10% of energy from added sugars. More specifically, 75% of children aged 2–11 years, with <10% of energy intake from added sugars, consumed no fruits or vegetables. Twenty five percent (25%) of the children consuming ≥10% added sugars also reported no fruit consumption and 75% consumed less than one vegetable portion per day (one fruit and one vegetable choice ~80 g in children). In comparison, half of the adolescents with <10% of energy from added sugars consumed one fruit portion and almost two vegetable portions, whereas those consuming 10% of added sugars had significantly lower fruit intake (*p* < 0.029).

Sensitivity analysis revealed that total sugar intake was 14.7% in under-reporters, 17.1% in plausible-reporters, and 14.3 % in over-reporters. Added sugar intake was 7.8%, 8.5%, and 8.3% respectively, with 31% of plausible- and 31.6% over-reporters consuming ≥10% of energy from added sugar. This was significantly lower among under-reporters with only 9.8% reporting added sugar intake ≥10% of total energy.

The likelihood of being overweight/obese was 2.33 times higher (95% CI: 1.298, 4.183, *p* = 0.005) for children and adolescents with total sugar consumption above the population median (13.6%) when specific food groups adjusted for energy were accounted for (Table 3; model 1), and 1.68 (95% CI: 1.008, 2.817, *p* = 0.047) when macronutrient intakes with respect to energy intake were accounted for (model 2). Children and adolescents consuming ≥10% of energy from added sugars were 2.57 times more likely (95% CI: 1.398, 4.717, *p* = 0.002) to be overweight/obese compared to those that consumed less <10% in model 1 and 1.77 time in model 2 (95% CI: 1.008, 3.096, *p* = 0.047). The results for the odds of overweight/obese when the median for added sugars was assessed, were very similar with those for ≥10%, as expected, as the median intake was 9.4%. In both cases the models were adjusted for level of physical activity, total screen time, sex, and parental educational level and professional status. The foods model was adjusted for total energy intake as well. Following the mixed effects logistic regressions, predictive probability of overweight/obesity, based on the models used, were derived.

**Figure 3:**
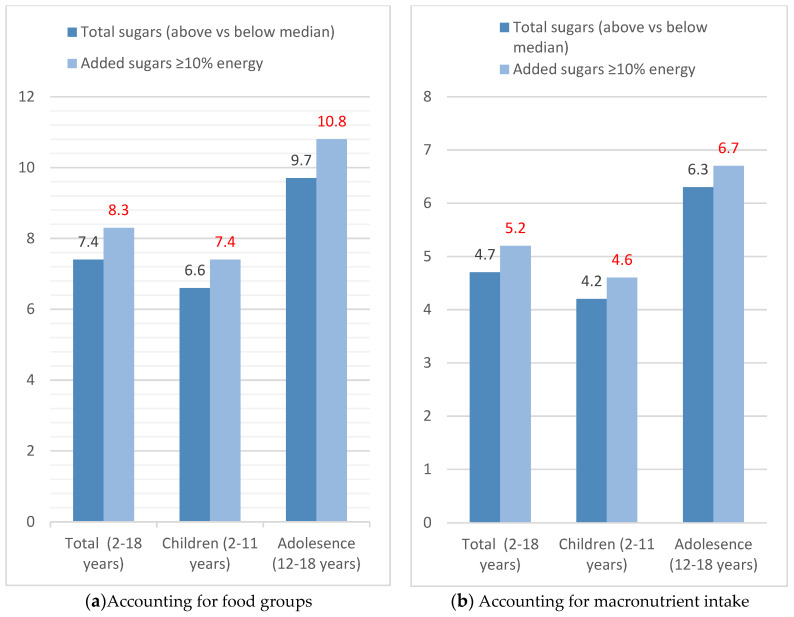
Predicted probability of being overweight/obese for children and adolescents by total- and added-sugar intake. (**a**) Accounting for food groups. (**b**) Accounting for macronutrient intake.; values in red represent predicted probability when consuming added sugars ≥10% of energy.

In Figure 3a,b, the predicted probabilities of being overweight/obese are depicted. In Figure 3a the probability increased significantly for higher total sugar and for ≥10% of added sugar intake, when accounting for specific food group intakes, as per Table 2. Specifically, consuming >10% of total energy from added sugar increases the probability of being overweight/obese by 8.3% in total sample (*p* = 0.002), by 7.4% in children (*p* = 0.003), and by 10.8% in adolescents (*p* = 0.005). The probability was lower for total sugar intake. In particular, it was 6.6% in children (*p* = 0.005), and 9.7% in adolescents (*p* = 0.008). When the model included macronutrients instead of food groups, the probability of being overweight/obese was lower in both cases compared to the first model (Figure 3b) but remained statistically significant.

## 4. Discussion

The present study examined total sugar and added sugar intakes, their major food contributors, and it assessed the relations between higher intake and likelihood of obesity in a representative sample of children and adolescents in Greece. The major finding of this study wasthat a high percentage of children and adolescents (20.6%) consumed added sugars above the recommended threshold of 10%, placing them at increased risk for overweight and obesity. It was also found that higher dietary total sugars intake was linked with higher added sugars, since approximately half of the amounts were derived from foods containing added sugars: sweets, processed grains, SSBs, and baked products. These findings included all children and adolescents assessed, excluding extreme over-reporters, to avoid biased errors in estimates. When under-reporters were excluded, which had a significant lower intake, this percentage reached 30%, further underlining the magnitude of added sugar intake with potential associated obesity risk. Specifically, the likelihood of being overweight or obese more than doubled for those with higher than recommended added sugar intakes and the predicted probability of becoming overweight/obese increased by 8.3%. If we were to extrapolate these findings to the total number of children and adolescents in Greece, it is estimated that 36 895 children and adolescents are likely to be or will probably become overweight and obese.

The definitions for sugars, including total and added, remains to be a challenge for researchers. The WHO On uses the term “free sugar” instead of “added sugar” in their sugar recommendations, which means that free sugar includes added and total sugars which are naturally present in fruit juices and fruit juice concentrates [37]. To overcome this problem, and account for all sugars but at the same time, distinguish the effects of added sugars, this study investigated total sugar as well as added sugar intakes. According to recent data, total sugar intake ranges from 17% to 34.8% in children and from 15.4% to 29.6% in adolescents worldwide [38]. Based on results from our study, total sugars were at the lower end of the worldwide ranges reported, but over half of total sugars were attained by addition. This was due to the fact that the majority of total sugars in this sample were derived from sweets, processed/refined grains and cereals, and SSBs, especially among adolescents, and fruit intake was very low. In addition, the median intake of added sugars among children and adolescents was close to the upper recommended intake, with one fifth of children and one fourth of adolescents exceeding it. These results are in agreement with an earlier study that reported high intake of added and naturally present simple sugars among school aged children [14]. In Canada the range of added sugars intake was estimated from 10% to 13% of total energy intake among different population subgroups, with higher intakes among adolescents [39], the latter being in agreement with the results of our study as well as results from a recent review of nationally representative surveys across the world [38]. This review reported that intakes of added sugars were higher among school aged children and adolescents, than in younger children.

It has been reported that higher added sugar intake is associated with lower diet quality [40,41]. In this study the majority of children and adolescents, irrespectively of added sugar intake, had poor dietary intakes as depicted by the median intakes of fruit, vegetables, fiber, and vegetable protein. This is in accordance with the results of other studies that found that even at lower deciles of added sugars intake, large percentages of children and adolescents had nutrient intakes below the average requirements [42], underlying poor dietary habits. Between studies controversies can be explained by differences in assessing the main foods that contribute to total and added sugar intake. In this population, other than sweets, processed/refined grains and cereals were the main contributors of total and added sugars, in both children and adolescents, foods that are usually enriched with many nutrients. Fruits including 100% fruit juices, although low in the majority of the population, also highly contributed, unlike drinks containing added sugars, that were at the lower end of food contributors.

The probability of overweight and obesity increased significantly with higher total sugar intake and higher added sugar intake above the recommendations. The potential effect of total sugar intake, irrespective of total energy consumption, was evaluated in relation to other food group intakes, granted that a recent systematic review suggested a diet with a lower percentage of obesogenic foods, including sugars, may reduce the risk of developing obesity among children [22]. Simple sugars overall have a high glycemic index, and lead to a faster and greater insulin secretion; a hormone that has an anabolic effect. This can be one major reason of the higher probability of overweight and obesity found in this study among children and adolescents with added sugar consumption above 10% of total energy in foods of low quality. The latter was reinforced by the fact that the likelihood of overweight and obesity was higher when the same model was adjusted for actual food intake, not only macronutrients, while maintaining total energy and other obesity confounders constant. To our knowledge, this is the first study that has compared the effects of food vs macronutrients on weight status, without seeking for specific dietary patterns but rather examining actual intakes of protective healthy foods and actual macronutrients. This information is important, as obesity is hard to treat [43], and although body weight can be modified by diet, treatments generally tend to have poor long term outcomes [44,45]. This is because obesity demands a more sophisticated approach than counting calories [44], including the understanding of pathophysiological and endocrine functions that occur post food consumption, reinforcing the results of this study. Furthermore, it has been reported that overconsumption of foods high in simple sugars replace more nutrient dense foods, and result in nutrient inadequacies [9,37]. This may be the case among children and adolescents in Greece based on a previous study reporting that most vitamins and essential minerals were derived from low-quality foods [46].

Study limitations include potential under-reporting of food-consumption, which could have led in an underestimation of energy, overall macronutrient intake, including total and added sugar intake. This said, based on the sensitivity analysis performed sugar intake can only be more but not less than the reported, since under-reporters had significantly lower consumption. Another limitation due to the cross-sectional nature of the study is that temporal relationship cannot be established; however, prediction probability analysis was used to estimate future trends, if levels of added sugars are not reduced and healthier dietary choices are not attained.

In conclusion children and adolescents need to develop healthy nutrition habits to maintain healthy body weight and prevent obesity development in adolescence and beyond. Interventions to prevent and control obesity focus on solutions which help acquire healthier behaviors [47]. Effort should be placed, not only to reduce added sugars, but to improve overall dietary intake in terms of foods consumed, emphasizing vegetables and plant-based protein. The results of this study confirm the strong recommendation of WHO for a reduced sugar intake, emphasizing on reducing intake to <10% of total energy intake for preventing and controlling excess body weight.

## Figures and Tables

**Table 1:** Anthropometric, dietary and lifestyle characteristics of children and adolescents, with main guardian sociodemographic information.

		Age Category		
	Total	Children	Adolescents	*p* Value
***n***	1165	781	384	-
**Weight, kg, mean (SD)**	40.7 (17.1)	31.9 (10.6)	58.6 (13.6)	<0.001
**Height, m, mean, (SD)**	1.44 (0.23)	1.33 (0.19)	1.66 (0.11)	<0.001
**BMI, kg/m2, mean, (SD)**	18.5 (3.3)	17.3 (2.4)	21.1 (3.4)	<0.001
Weight status *, *n* (%)				<0.001
Healthy weight	1000 (85.8)	699 (89.5)	301 (78.4)	
Overweight	142 (12.2)	66 (8.5)	76 (19.8)	
Obese	23 (2.0)	16 (2.1)	7 (1.8)	
**Total sugar**				
Grams, median (IQR)	43.7 (32.5, 70.0)	41.2 (41.2, 66.8)	55.7 (38.1, 80.6)	<0.001
Total calories from total sugars, median (IQR)	175 (165, 280)	165 (165, 267)	222 (153, 319)	<0.001
Total % energy intake, mean, (SD)	15.6 (6.7)	16.3 (6.3)	14.3 (7.4)	<0.001
**≥median, *n* (%)**	587 (41.8)	303 (38.8)	184 (47.9)	0.003
**Added sugar**				
Total grams, median (IQR)	28.4 (17.7, 34.5)	28.4 (22.7, 28.4)	24.5 (13.1, 44.6)	<0.001
Total calories from added sugar, median (IQR)	114 (71, 138)	114 (91, 114)	98 (52, 178)	<0.001
Total % energy intake median (IQR)	9.4 (4.5, 9.4)	9.4 (6.3, 9.4)	5.5 (3.5, 9.8)	<0.001
≥median, *n* (%)	259 (22.2)	156 (20.0)		0.008
≥10% of total energy, *n* (%)	240 (20.6)	146 (18.7)	94 (24.5)	0.022
**Total METS, median (IQR)**	0 (0, 3953)	0 (0, 2016)	3228 (1477, 6628)	<0.001
**Total screen time (hours), median 1 (IQR)**	3 (1.5, 3.75)	3.75 (1.6, 3.75)	2.45 (1.3, 4.0)	<0.001
**Primary guardian Educational level, *n* (%) **				<0.001
≤6 years	29 (3.11)	8 (1.2)	21 (7.6)	
>6–12 years	599 (64.2)	457 (69.6)	142 (51.5)	
≥12 years	305 (32.7)	192 (29.2)	113 (40.9)	
**Primary Guardian Professional Status, *n* (%)**				<0.001
Employed	727 (78.8)	561 (86.0)	166 (61.3)	
Unemployed/Homeworkers	157 (17.0)	86 (13.2)	71 (26.2)	
Pension	30 (4.2)	5 (0.8)	34 (12.6)	

Significance level set at 5%; Student *t*-test for normally distributed values and Kruskal–Wallis test for skewed numerical variables (two group comparison); chi square test or Fisher’s exact test for categorical variables. * Weight status categorized based on IOTF criteria. ^1^ Total screen time includes total TV-viewing, computer, phone, and other screens that do not involve active movement. IQR: Interquartile range.

**Table 2:** Estimated mean energy, macronutrient, and selected food group intake in children and adolescents by added sugar intake status.

	Food Group Intake Per Day	Added Sugars Intake Status		Significance
		<10% Total Calories	≥10% Total Calories	*p* Value
**Children (2–11 years)**	**Food groups (median; IQR)**			
	Fruit, gr	0 (0, 116)	71.3 (0, 166)	<0.001
	Vegetables, gr	0 (0, 71)	55 (14, 96.2)	<0.001
	Animal protein, gr	53 ^1^	54 (22, 86)	<0.001
	Non milk dairy, gr	30 ^1^	20.1 (0, 46.5)	<0.001
	**Energy, kcal**	1378 (571)	1692 (777)	<0.001
	**Macronutrients, % Energy**			
	Non sugars carbohydrates	29.8 (6.3)	27.0 (8.9)	<0.001
	Total sugars	14.6 (4.3)	23.9 (8.1)	<0.001
	Animal protein	7.5 (7.5, 7.6)	7.1 (4.7, 10.3)	<0.001
	Vegetable protein	4.1 (1.2)	4.2 (1.8)	0.514
	Total Fat	40.1 (5.8)	36.7 (7.0)	<0.001
	Total Fiber	7.1 (7.1, 9.9)	10.7 (7.5, 16.5)	<0.001
**Adolescents (12–18 years)**	**Food groups (median; IQR)**			
	Fruit, gr	87 (0, 193)	4 (0, 147)	0.029
	Vegetables, gr	138 (59, 254)	110 (41, 199)	0.135
	Animal protein, gr	98 (52, 183)	59 (23, 106)	<0.001
	Non milk dairy, gr	30 (11, 73)	23 (7, 60)	0.180
	**Energy, kcal**	1862 (749)	1799 (780)	0.484
	**Macronutrients, %Energy**			
	Non sugars carbohydrates	29.4 (8.3)	29.3 (10.0)	0.889
	Total sugars	12.2 (6.5)	20.7 (6.0	<0.001
	Animal protein, %Energy	9.0 (5.5, 11.8)	6.4 (4.1, 10.5)	<0.001
	Vegetable protein, %Energy	4.0 (2.0)	4.2 (2.0)	0.607
	Total Fat, %Energy	42.1 (9.0)	37.7 (9.6)	<0.001
	Total Fiber, gr/day	12.9 (8.9, 18.3)	13.1 (9.4, 18.2)	0.726

^1^ no IQR differences. Skewed variables are summarized using median and IQR, whereas normally distributed variables with mean (±sd). Group comparisons have been made by Student *t*-test vs Kruskal–Wallis test when skewed. Animal protein food group includes all meat, eggs, and seafood/fish. Vegetables include all starchy, non-starchy vegetables and potatoes. Fruit group includes fruit and 100% fruit juice. Non-milk dairy includes yogurt and cheese. Plant protein was not included in food groups, due to limited consumption. Non sugars carbohydrates include total carbohydrate intake after subtracting total sugar intake, and then expressed as %energy intake.

**Table 3:** Odds of overweight/obesity in total sample using 2 models: (a) food groups and (b) macronutrients.

	Model 1			Model 2		
	Odds Ratio	95% Conf.Interval		Odds Ratio	[95% Conf.Interval]	
Total Sugar, % energy (above vs below median)	2.33	1.298	4.183	1.69	1.008	2.817
Added Sugars, % energy (above vs below median)	2.64	1.459	4.789	1.80	1.046	3.124
Added Sugars ≥10% energy	2.57	1.398	4.717	1.77	1.008	3.096

Results following mixed effects logistic regression; for total sugars population median was used; reference population were children <50th percentile. Model 1 includes: animal- and plant- protein, non-milk dairy, fruit, and vegetable intakes, adjusted for energy; Model 2 includes: macronutrient intakes (fiber, fat, protein, other non-sugar carbohydrates). Both models were adjusted for children’s and adolescent’s activity level (IPAQ), total screen time and for the primary guardian’s educational level and professional status. Significance at α = 5%.

## Supplementary Materials

The following are available online at https://www.mdpi.com/article/10.3390/children8080676/s1, Supplementary Table S1: Grouping of primary food groups for specific study purposes.
