# Supplementary material for: Dietary Sugar Intake and Its Association with Obesity in Children and Adolescents
Source: Children (Basel). 2021 Aug 3;8(8):676. doi: 10.3390/children8080676 (PMC8391470; doi:10.3390/children8080676)
Supplement: Supplementary file 1 [file children-08-00676-s001.zip › children-1275195-supplementary.pdf]

Supplementary Table S1: Grouping of primary food groups for specific study purposes

Original grouping	Modified grouping
**Fruits**	Fruit group
Fresh fruits, cooked or dried	
Fruit juice	
Natural fruit juices unsweetened	
**Non-starchy vegetables**	Vegetable group
Green leafy vegetables	
Tomatoes, carrots, lettuce	
Mixed and other vegetables	
Vegetable juice	
Starchy vegetables	
Corn, beans, green beans	
Pumpkin	
Sweet potatoes	
Potatoes	
**Wholegrain cereals**	Wholegrain cereals
Wholegrain - crudely processed cereal products	
**Processed cereals**	Refined cereals
Processed grain & cereal products including breakfast cereals	
**Legumes**	Plant protein
Legumes (i.e., beans)	
Meat alternatives, soy, tofu	
**Nuts**	
Nuts, almonds, seeds	
Peanut butter	
Almond milk	
Milk and milk drinks low in sugar	Milk
Yogurt	Non milk dairy
**Cheese**	
Eggs	Animal protein
**Fish and Shellfish** (including fresh and frozen)	
Shellfish	
**Red meat** (Lamb, pork, veal, game)	
**White meat** (poultry)	
**Processed red meat** (Sausages, ham, salami, beacon of red meat origin)	Processed animal protein
**Processed white meat** (Sausages of white meat origin, Chicken nuggets)	
**Processed fish** (Smoked, canned and salted fish)	
Fish sticks	
**Olive oil and Olives**	Olive oil
**Vegetable fat**	Other oils
Vegetable oils, vegetable fat, vegetable oil-based salad dressing	

**Animal fat**	All other fats
Butter, mayonnaise	
White sauce, cream	
**Sugar-sweetened beverages**	SSb's
Carbonated and non-carbonated sugar-sweetened beverages	
**Desserts and Sweets**	Sweets
**Sweets, candy, chocolate**	
**Milk desserts**	
**Sugary foods (i.e., baklavas)**	
**Sugar**	
Sugar, honey, syrup	
**Baked products**	
Cake, biscuit, pie, muffin, doughnuts	
**Fast-Food**	Fast food
Pizza, souvlaki, burger, fried potatoes	
**Baby food**	Baby food
